# RNASeq profiling of UTR expression during neuronal plasticity

**DOI:** 10.1186/1471-2105-13-S12-A4

**Published:** 2012-07-31

**Authors:** Benjamin J Harrison, Robert M Flight, Abdallah Eteleeb, Eric C Rouchka, Jeffrey C Petruska

**Affiliations:** 1 Department of Anatomical Sciences and Neurobiology, University of Louisville, Louisville, KY 40292, USA; 2 Kentucky Spinal Cord Injury Research Center, Department of Neurological Surgery, University of Louisville, Louisville, KY 40292, USA; 3 Department of Computer Engineering and Computer Science, University of Louisville, Louisville, KY 40292, USA

## Background

Neurons interact with, and are influenced by, tissues that are remote from the cell body. For example, sensory neuron cell bodies are located within peri-spinal ganglia but are connected to both the spinal cord and skin via their axons projecting through dorsal roots and peripheral nerves. Biochemical signals from anatomical compartments (spinal cord / root / ganglion / nerve / skin) modulate the molecular biology of neurons which can respond to signals from any/all of these remote regions. One mechanism by which neurons respond to these signals and interact with their targets is by actively transporting mRNA to that region. There, the mRNA is translated to produce protein at locally-determined positions and times. A growing body of evidence shows that untranslated regions (UTRs) of genes are important for this targeting. For example, 3’-UTRs contain 50nt “zip code” consensus binding sites for cis-acting zip code-binding proteins (ZPBs) that drive axonal targeting of mRNA [1]. We therefore hypothesized that gene expression during collateral sprouting, an axonal growth process that is highly responsive to target-derived factors, might involve differential regulation of UTR components.

## Methods

Axonal collateral sprouting of sensory neuron axons was induced and progressed for 7 or 14 days. RNA was then harvested from sensory ganglia of experimental and control animals for total transcriptome sequencing using the Illumina platform. Sequence reads were aligned to the currently available reference Rat genome (rn4), and all known exons (coding and untranslated) were tested for differential expression at each time-point compared to those from control ganglia. We also identified the subset of genes for which the sprouting-associated expression change was due to regulation of the UTR while the coding region of that gene was unchanged between sprouting and control. Thus, we identified the genes whose sprouting-associated regulation was due to changes in expression of the UTR and not of the coding sequence (CDS). These genes with differentially-expressed UTRs and non-regulated coding regions were then tested for over-representation of functional classes (Gene Ontology) and for known RNA binding protein binding sites.

## Results

Ontology enrichment analysis indicates that UTR-specific regulation of expression of numerous genes may be important for neuronal plasticity responses to target-derived factors, and local regulation of axonal plasticity.
